# THE EARLY LIFE CONTEXTS OF THE HEALTH AND RETIREMENT STUDY HCAP SAMPLE: NEW INSIGHTS INTO DEMENTIA RISK FACTORS

**DOI:** 10.1093/geroni/igad104.3632

**Published:** 2023-12-21

**Authors:** Jacqui Smith, Marina Larkina, Wenshan Yu

**Affiliations:** University of Michigan, Ann Arbor, Michigan, United States; University of Michigan, Ann Arbor, Michigan, United States; University of Michigan, Ann Arbor, Michigan, United States

## Abstract

There’s increasing interest in identifying potentially modifiable early-life socioenvironmental factors associated with late-life cognitive decline and dementia. To date, much research points to the importance of the number of years spent in education and age. We sought to extend evidence for potential modifiable targets by examining life history data available to date for women and men in the 2016 HRS-HCAP sample (N = 2645; Mean Age = 76; 61% women). The life history data were drawn from three HRS public cross-wave files: the 2015-2017 Life History Mail Surveys, the 1992-2018 Childhood Family and Childhood Health file, and the 1992-2020 Geographical Mobility File. Among the early factors examined were the US Census region of birth and school location at age 10; childhood physical and mental health conditions; childhood family SES; early learning and sensory/motor problems, early life activity engagement (cognitive, physical, social), college majors, and post-education work. The HRS-HCAP algorithm classifications (Dementia, MCI, vs Normal Cognition) served as outcomes. In addition to quantity of education and age/cohort, significant effects (all ps < .001) were revealed for childhood geographical locations, childhood mental health, and early-life access to and engagement in enriching activities. Over time, whereas the impact of changes such as increased access to post-secondary education and enriching high school activities are observed, other modifiable factors emerge. Tracking the impacts on cognitive aging of historical changes and disparities in the life course contexts of people in the HRS-HCAP subsample opens a new window into relevant social-environmental factors to target for intervention

